# Sealing versus partial caries removal in primary molars: a randomized clinical trial

**DOI:** 10.1186/1472-6831-14-58

**Published:** 2014-05-28

**Authors:** Daniela Hesse, Clarissa Calil Bonifácio, Fausto Medeiros Mendes, Mariana Minatel Braga, José Carlos Pettorossi Imparato, Daniela Prócida Raggio

**Affiliations:** 1 Orthodontics and Pediatric Dentistry Department, Dental School, University of São Paulo - USP, Av. Lineu Prestes, São Paulo, SP 2227, Brazil; 2 Department of Conservative Dentistry, Academic Centre for Dentistry Amsterdam - ACTA, Amsterdam, The Netherlands

**Keywords:** Pit and fissure sealants, Dental caries, Primary teeth, Caries lesion, Partially excavation

## Abstract

**Background:**

The resin-based pit and fissure sealant is considered a successful tool in caries prevention, however there is a growing evidence of its use in controlling already established caries in posterior teeth. The aim of this clinical trial is to verify the efficacy of pit and fissure sealants in arresting dentinal caries lesions compared to partial excavation and restorative treatment in primary molar teeth.

**Methods:**

Thirty six patients with occlusal cavitated primary molar reaching outer half of dentin were selected. The patients were randomly allocated into two groups: sealant application (experimental group – n = 17) and restoration with composite resin (control group – n = 19). Clinical and radiograph evaluation were performed after 6, 12 and 18 months. The chi-square test was used to verify the distribution of characteristics variables of the sample among the groups. The survival rate of treatments was evaluated using Kaplan–Meier survival and log-rank test. Fisher’s Exact and logistic regression tests were calculated in each evaluation period (α = 5%).

**Results:**

The control group showed significantly better clinical survival after 18 months (p = 0.0025). In both groups, no caries progression was registered on the radiographic evaluations.

**Conclusions:**

Sealing had similar efficacy in the arrestment of caries progression of cavitated occlusal lesions compared to partial excavation of the lesions, even though the frequency of re-treatments was significantly higher in sealed lesions.

**Trial registration:**

Registro Brasileiro de Ensaios Clínicos (ReBEC): RBR-9kkv53

## Background

Dental caries is a disease with identified etiology and able to be prevented and controlled [1,2]. It is established that caries development is dependent on the biofilm stagnation area on the dental surface. The metabolic activity in dental plaque is the fuel for caries lesion development and the demineralization occurs as a result of this dynamic process [3]. Therefore, a lesion could be arrested by controlling the biofilm on its surface. However, it is more difficult to enhance and disorganize the biofilm in cavitated lesions, even with careful brushing.

Conventional caries lesions management is usually based on operative procedures to re-establish the surface integrity and enable efficient dental plaque removal [3]. Resin-based sealants were developed to be applied on the occlusal surfaces susceptible to the development of caries lesions, covering the pits and fissures, creating a layer that avoids the retention of food debris and biofilm in these areas, thus preventing the development of caries lesions [4]. There is a strong evidence of the benefits in using sealants as a preventive approach [2,5-7], and studies have been conducted using sealants for the treatment of caries lesions, with results showing that while the sealant remains adhered to the tooth surface, the lesion is arrested [8-11].

There is considerable evidence that the complete removal of infected dentin is not required to achieve caries lesions arrestment [12], therefore the partial removal of caries lesion and restoration with composite resin can be considered good clinical practice [13]. Indeed, this statement is supported by the results of clinical trials, which reported success rates of 100% after 6 months [14] and 3 years of follow-up [11]. The application of resin-based sealants have also shown high success rates, ranging from 80% to [11,15] 94.8% [4] after 2 and 3-year follow-up in permanent molars and 61% [16,17] to 88% [18] after 2 and 3 years of assessment in primary molars.

The caries-preventive effect of pit and fissure sealants has been intensively studied in the literature and shows strong evidence of its effectiveness [2,5,19]. However, the indication for applying occlusal sealants seems to be shifting from primary prevention to a therapeutic decision for caries management in enamel and outer half of the dentin lesions [20]. The fact that caries lesion can be arrested by sealing the cavity is not recent. Since the 1970s some researches highlighted this fact. The idea of sealing caries lesions without invasive intervention possibly began with Handelman et al. [21]. Since then, a large number of studies have been developed by using the resin-based sealants over caries and the results suggest that the lesion is arrested [8-11,21-25].

To the best of our knowledge there is one study that has investigated the application of resin-based fissure sealant for the treatment of caries lesions in primary molars [18], but they have worked only with non-cavitated lesions. Since there are several difficulties in the management of children’s behavior during conventional restorative treatment, sealing dentinal caries lesions in primary teeth can be an interesting and less invasive alternative. The hypothesis tested was that more caries progression is expected with the application of a resin-based pit and fissure sealant compared to partial excavation and restorative treatment. Therefore, the aim of this clinical study is to verify the efficacy of pit and fissure sealants in arresting dentinal caries lesions compared to partial excavation and restorative treatment in primary molar teeth.

## Methods

### Participants and recruitment

After approval from the Local Research Ethics Committee (protocol 204/05 – School of Dentistry, University of São Paulo), thirty six children seeking dental treatment at the School of Dentistry of University of São Paulo were selected and written consent was obtained from all legal guardians. The study was performed during the period of 2007 until 2011. The children were aged 4 to 9 years old (mean age = 7). Sample power was calculated (using an α error of 5%) and resulted in 0.9.

The children were selected after clinical and radiographic examination by the operator (DH), who was trained and calibrated for the caries assessment according to ICDAS scores [26] and for caries activity according to the visual-tactile criteria of Nyvad [27]. Lesions with a matt and rough surface were scored active, whereas lesions with a shiny and smooth surface were scored inactive. During the first visit, the children had the surfaces of teeth professionally cleaned with pumice and rotating brushes, and air-dried before assessment of caries lesion presence [26] and activity [27]. Also, bitewing radiographs were taken during this first appointment, in order to evaluate the deepness of the caries lesion.

The caries experience of patients included in this study was classified as above average (mean = 6.0) according to age-related dmft values (decayed, missed, filled teeth) for Brazilians [28].

Only children with good general health, and at least one primary molar with occlusal active caries lesion [27] classified as ICDAS score 5 [26], with an opening not wider than 3 mm diameter in the enamel, measured with a millimeter probe, and with no pain history were included in this study. Radiographically, the lesion should reach the dentin, but be maximally limited to the half way through this substrate. Moreover, the selected tooth could not have any restoration or caries lesions in surfaces that could interfere with the study proposal.

Baseline bitewing radiographs were taken using bitewing holders (Indusbello, Londrina, Brazil). The equipment used was a Spectro 70X (Dabi Atlante, Ribeirão Preto, Brazil) with 70 kV, 8 mA and the exposure time was 0.4 seconds. The films used were speed group E (Eastman Kodak Co., New York, USA) and they were manually developed using standard processing times.

The patients were randomly allocated into two groups with the use of a list of random numbers generated by computer:

• Experimental Group: pit and fissure resin-based sealant application, without removing caries tissue (n = 17).

• Control Group: restorative treatment with composite resin, after partial dentinal caries removal (n = 19).

All treatments were performed by one operator properly trained and helped by a dental assistant. The operator was a final-year undergraduate dental student who was previously trained to perform both techniques used in this study. A training week was included to give the operator the opportunity to familiarize herself with the sealants application and restorative technique prior to the start of the operative phase.

### Intervention

Only one tooth per child was included in the research. If more than one cavity met the inclusion criteria, one of them was randomly chosen. The other caries lesions in the selected children were treated by the researchers, who also provided information regarding diet and oral hygiene instructions.

The teeth from the experimental group were sealed according to the following protocol: (a) occlusal surface cleaned with pumice; (b) local anesthesia applied; (c) rubber dam applied; (d) 37% phosphoric acid placed on occlusal surface for 15 seconds; (e) surface rinsed and dried; (f) adhesive system (Adper Single Bond 2, 3 M ESPE, Saint Paul, USA) applied, following the manufacturer’s instructions and light cured for 20 seconds; (g) resin-based sealant (Fluroshield, Dentsply, Rio de Janeiro, Brazil) applied and light cured for 20 seconds; (h) occlusion checked and adjusted when necessary.

The teeth from the control group were restored according to the following protocol: (a) occlusal surface cleaned with pumice; (b) local anesthesia applied; (c) rubber dam applied; (d) cavity opened in enamel with a diamond bur in high speed, caries lesion completely removed in the enamel/dentin junction, and dentinal caries lesion partially removed with hand instruments until the dentin started to become ‘firm and leathery’ [29]; (e) 37% phosphoric acid applied in the cavity for 15 seconds; (f) surface rinsed and dried; (g) adhesive system (Adper Single Bond 2, 3 M ESPE, Saint Paul, USA) applied, following the manufacturer’s instructions and light cured for 20 seconds; (h) restoration with composite resin (Z250, 3 M ESPE, Saint Paul, USA), using the incremental technique until cavity was filled and light cured of each increment for 20 seconds; (i) occlusion checked and adjusted when necessary.

### Evaluation

The follow-up period involved the evaluation of patients at 6, 12 and 18 months after treatment and three examiners were responsible for the assessments. One of the examiners performed the clinical evaluations, while the other two carried out the radiographic evaluation.

The marginal integrity of sealants and restorations were assessed clinically by one examiner trained by a “golden standard” evaluator regarding the evaluation criteria [30,31]. In order to calculate the intra examiner concordance, 15 patients involved in the research were re-evaluated with an interval of two weeks (kappa intra value = 1.00). The scores for clinical assessment were: partial loss and total loss (failure) or total retention (success). When integrity failures were found during the follow-up visits, the re-application of the sealant or restoration-repair was done, however the related tooth was considered as a failure in the subsequent clinical analysis. The evaluation criteria for the clinical assessment at the follow-ups were the same for both groups.

Two trained and calibrated evaluators specialists in pediatric dentistry, who underwent four hours of specific training with radiographs, provided by a “gold standard” examiner, evaluated the caries lesion progression radiographically (kappa inter value = 0.85). The outcome variable was the caries progression status (absence or presence of caries progression). The examiners assessed the radiographs through paired evaluations comparing two by two, blinded regarding chronological order of the radiographs and without the aid of any magnification loops. In cases of disagreement between the evaluators, a third examiner made the final decision.

### Statistical analysis

All data were recorded in individual forms. The chi-square test was used to verify the distribution of characteristics variables of the sample among the groups. Fisher exact test was used to analyze the statistical differences in clinical retention and radiographic changes between the groups in the three follow-up assessments. The survival rate of each treatment was estimated by Kaplan-Meier analyses. The logistic regression test was used to evaluate the association among characteristics variables and the outcome. The level of significance considered for all tests was 5%.

## Results

Of the 36 children (mean age = 7 years) who participate in the study, 16 (44.4%) were female and 20 (55.6%) were male. The children were randomly allocated, and 17 (47.2%) of them had their cavities sealed, without removing caries tissue and 19 (52.8%) received restorative treatment with composite resin, after partial dentinal caries removal was performed; 14 (39.9%) were placed in the upper jaw and 22 (61.1%) in the lower jaw; 19 (52.8%) were in the left side and 17 (47.2%) on the right side; 11 (30.5%) were first primary molars and 25 (69.5%) were second primary molars. The chi-square test showed an equal distribution in both groups with regards to the characteristics variables of the sample (Table 1) (p > 0.05).

**Table 1:** Distribution of the participating children according to treatment protocols

	***Age (yrs)***		***Gender***		***Jaw***		***Jaw side***		***Molar***	
	***Mean***	***Range***	***Boys (n)***	***Girls (n)***	***Upper (n)***	***Lower (n)***	***Right (n)***	***Left (n)***	***1***^***st ***^ ***molar (n)***	***2***^***nd ***^ ***molar (n)***
***Sealant application***	6.7	4-9	9	8	4	13	11	6	7	10
***Restorative treatment***	7.3	4-9	11	8	10	9	8	11	4	15

Chi-square test showed an equal distribution in both groups with regards to the characteristics variables of the sample (p > 0.05).

*n* number of children.

**Figure 1:**
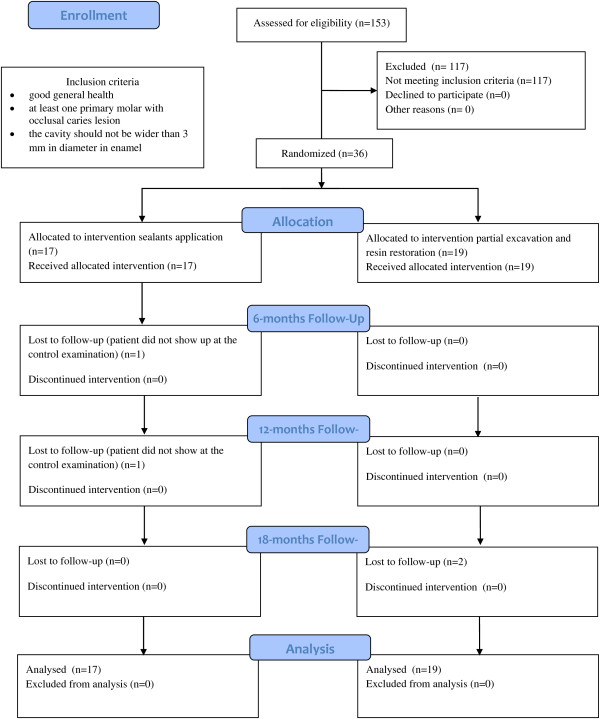
**Consort flow diagram of the trial.***n* number of children.

A CONSORT flow diagram (Figure 1) shows: number of children, number of sealants and restorations, number of presence or absence of patients at the three evaluation times. The results of the clinical and radiograph assessments from both groups are expressed in Table 2. After 18 months, only 2 drop-outs in the control group is registered. However, two children in the experimental group did not show up at the 6- and 12-month control examinations but were examined at the 12- and 18-month examination, respectively. Therefore, both patients were re-included in the study. A significant difference was found after 18 months with better results regarding the clinical examination for the control group. After 6, 12 and 18 months, none of the lesions in both groups showed progression (Table 2).

**Table 2:** Clinical and radiographic results after 6, 12 and 18 months assessments

	**Clinical assessment**				**Radiographic assessment**	
	***n***	***Success***	***Failure***	***p***	***Absence of caries progression***	***Presence of caries progression***
***Sealant application***						
6 months	16	14 (87.5%)	2 (12.5%)	0.23	16 (100%)	0
12 months	16	12 (75.0%)	4 (25.0%)	0.05	16 (100%)	0
18 months	17	11 (64.7%)	6 (35.3%)	0.0025*	17 (100%)	0
***Restorative treatment***						
6 months	19	19 (100%)	0		19 (100%)	0
12 months	19	19 (100%)	0		19 (100%)	0
18 months	17	17 (100%)	0		17 (100%)	0

*Significance tested by Fisher’s exact test.

No statistical analysis was performed for radiographic assessment because there were no cases of caries progression in both groups.

*n* number of children.

**Figure 2:**
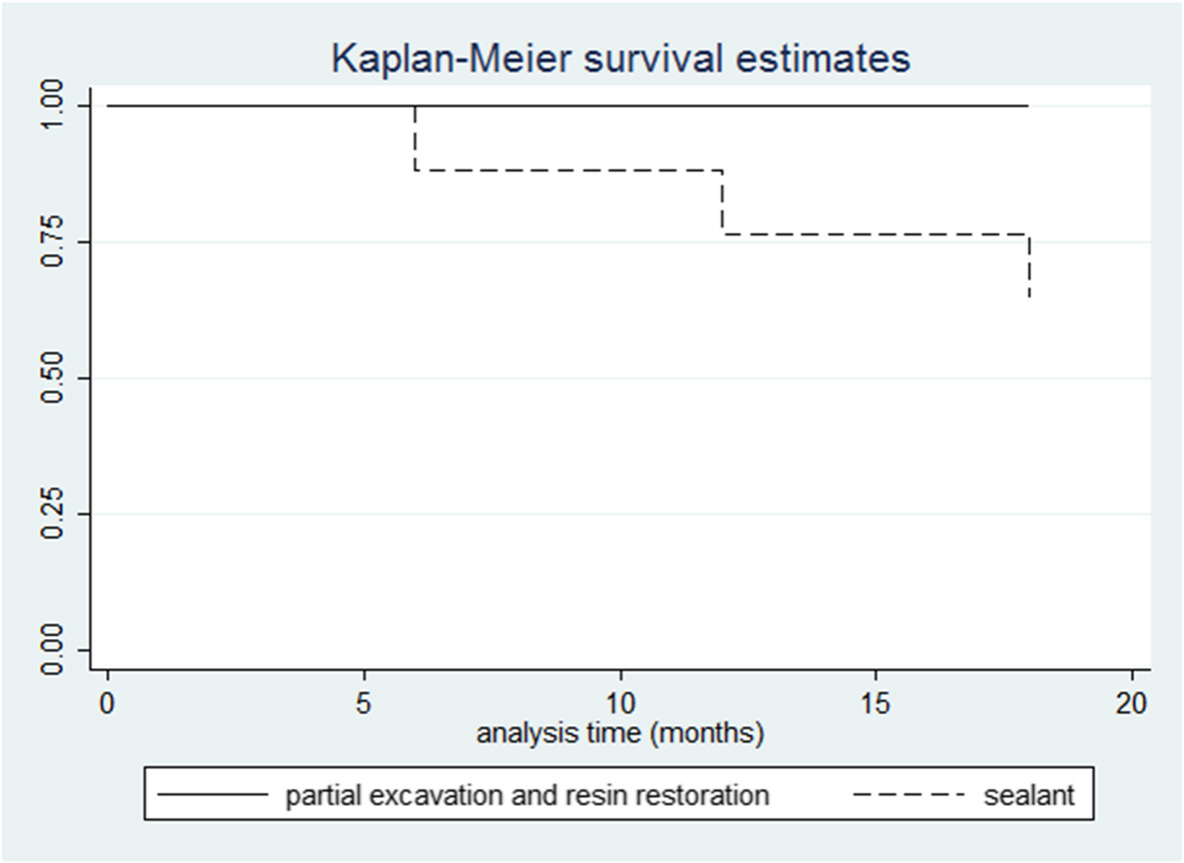
**Kaplan-Meier survival curves for the clinical evaluation.** Log-rank, p = 0.0053.

The logistic regression test was applied to test the association among patient’s characteristics variables and the outcome, and showed no association between any variable tested (gender, age, 1^st^ or 2^nd^ molar, upper or lower jaw and left or right side) and sealant failure.Figure 2 shows Kaplan–Meier survival estimates curve in which a higher longevity of composite resin restorations (control group) can be observed, when compared to sealant application (experimental group). Log rank test indicated a significant difference between the groups (p = 0.0053).

## Discussion

The hypothesis of this study stated that more caries progression in the experimental group was expected; however this hypothesis can be rejected due to the results (Table 2). Thus, over 18 months there is no difference in caries progression irrespective of caries removal. The lack of caries progression observed in the sealed teeth may be attributed to the fact that all molars presenting sealant failure were re-sealed, enabling the patient to control the biofilm and consequently arresting the caries progression [32]. Sealing may be an effective approach for treating cavitated occlusal caries with radiographic lesion penetration into the outer half of the dentin in primary molars; however, the results should be taken with caution because the sample size is limited in both experimental and control group.

In the same way as observed for the sealed teeth, the restored teeth showed caries arrestment. This was, in fact, expected, since the lesion was also isolated from the biofilm formation by restorative material. However, there is a risk of removing sound tooth substrate, when a tooth is treated by a restorative approach, while, the procedure for sealing is much less invasive. Furthermore, sealing dental caries offers the advantage of being less time-consuming than conventional restorative procedures. For this reason, sealing caries lesions in primary molars might be beneficial in treating non-cooperative children.

Another point of discussion is the use of adhesive system prior sealant application. This approach can be an alternative to increase the adhesion [33] and decrease microleakage in case of cavitaded occlusal lesions [34-38], as well as decline clinically the risk of failures of the sealants, especially in occlusal surfaces [35] and enhance the retention and longevity of this material [39]. In our study we used a conventional adhesive system prior sealant application in order to achieve better retention of the material in caries lesions that reached dentin, a more humid substrate that could negatively influence the retention results.

In our study, the teeth that had partial loss of the material had the occlusal surface re-sealed. The re-treatment was performed due to Ethical reasons; however as there were no cases of caries progression, these teeth were considered in subsequent radiograph evaluations. According to Handelman et al. [23], the treatment with sealants is efficient while the material is adhered to the tooth surface and the follow-up of teeth treated with sealants involves clinical and radiograph evaluation to assess the marginal integrity of the material and the arrestment of the lesion. Yet, according to Bakhshandeh et al. [11], the application of resin-based sealants on occlusal dentin lesions can postpone and even avoid conventional excavation and restoration of these lesions, as long as the sealant is intact and tight to the tooth. In these cases, even though the conventional restorative treatment may be necessary in the future, the prognosis for the individual tooth will be increased due to the postponement of a more invasive approach.

Nevertheless, we acknowledge the limits of our study as having a small sample size and a limited time of evaluation; however according to Hackshaw [40] there is nothing wrong with conducting well-designed small studies; they just need to be interpreted carefully. Yet, in our study sample power was calculated and resulted in 0.9, which is considered as a strong effect. As the effect size is based on the difference between the means, it is expected that for a greater effect, the sample size required will be smaller. As this study was conducted on a sample with high caries experience, and the study period was only 18 months, its value in terms of external validity might be limited. Nevertheless, the arrestment of lesions was observed in 100% of cases in a population with high experience of caries, so it might also result in caries arrestment in a population with average caries experience, and for longer times. But this hypothesis needs to be confirmed.

Systematic reviews [41,42] concluded that there was no evidence to justify the complete caries lesion excavation and partial caries tissue removal was recommended, including the maintenance of infected dentin in cases whose removal would increase the risk of pulp exposure. Thus, both systematic reviews available in the literature support the hypothesis that there is no need for complete removal of caries dentin towards the pulp. In our study, the control group was characterized by the partial caries removal and in all cases we observed arrested lesion. For that reason, our results support the theory that biofilm is responsible for caries lesions progression [3]. Therefore, the clinician should have in mind that restorative treatment aim to provide an adequate filling, giving the patient conditions to remove biofilm which leads to caries lesion arrestment [43]. Systematic reviews focused on sealing pit and fissures do not include caries lesions in dentin [2,5-7]. On the other hand, several studies have shown that by sealing the occlusal surface, it is possible to arrest dentinal caries [8-11,21-25]. However, most of these studies were performed in permanent teeth and the simple extrapolation of results obtained in previous studies with permanent teeth is not appropriate, since the primary teeth have individual physical and structural characteristics. The enamel in primary teeth is thinner and less mineralized than in permanent teeth, therefore the primary teeth has a higher rate of caries progression [44]. Based on our results, we can infer that there is no difference in caries progression when caries lesions in the outer half of dentin of primary teeth were treated with composite resin restoration or with resin-based sealant. Based on this, we can assume that there is no need of any caries tissue removal for arresting cavitated lesions located in the outer half of dentin in primary teeth.

## Conclusion

In conclusion, although the control group has presented less restoration failures, both treatments are similar in arresting the caries progression.

## Competing interests

The authors declare that they have no competing interests.

## Authors’ contributions

DH wrote the study protocol, performed literature review, clinical treatments and supervised evaluations and wrote the first draft of the manuscript. CCB made contributions during study protocol writing, supervised clinical treatments and made manuscript revisions, FMM performed the statistical analysis and made contributions to the interpretation of the results and manuscript revisions, MMB made contributions to the interpretation of the results and manuscript revisions, JCPI made contributions during study protocol writing, supervised clinical treatments and evaluations, DPR made contributions during study protocol writing, literature review, supervised clinical treatments and evaluations, made contributions to statistical analysis, as well as to the interpretation of the results and manuscript revisions. All authors have read and approved the final version of the manuscript.

## Pre-publication history

The pre-publication history for this paper can be accessed here:

http://www.biomedcentral.com/1472-6831/14/58/prepub
